# Evaluating an Abbreviated Version of Mindfulness-Based Cognitive Therapy Delivered via Telephone or Videoconferencing Compared to Enhanced Usual Care—Treatment for Migraine and Mood (TEAM-M) Study: Protocol for a Three-Arm Multisite Randomized Controlled Feasibility Trial

**DOI:** 10.2196/93627

**Published:** 2026-06-05

**Authors:** Chelsea J Siwik, Hallie Tankha, Jacob D Hill, Pallavi Visvanathan, Devyn Gaskins, Marisa DeSciscio, Narissa McCarty, Hannah O’Brien, Marissa Salvaterra, Cuiling Wang, Richard Lipton, Rebecca E Wells, Elizabeth Seng, Amanda J Shallcross

**Affiliations:** 1 Cleveland Clinic Lerner College of Medicine, Cleveland, OH, United States; 2 Department of Wellness and Preventive Medicine, Cleveland Clinic, 2050 East 96th Street, Cleveland, OH, 44106, United States, 1 216-448-4325; 3 Manhattan Center for Mindfulness-Based Therapy, New York, NY, United States; 4 Department of Public Health Sciences – Social Sciences and Health Policy, Wake Forest University School of Medicine, Atrium Health Wake Forest Baptist, Winston-Salem, NC, United States; 5 Saul R. Korey Department of Neurology, Albert Einstein College of Medicine, Bronx, NY, United States; 6 Department of Epidemiology & Population Health, Albert Einstein College of Medicine, Bronx, NY, United States; 7 Department of Neurology, Wake Forest University School of Medicine, Atrium Health Wake Forest Baptist, Winston-Salem, NC, United States; 8 Ferkauf Graduate School of Psychology, Yeshiva University, Bronx, NY, United States; 9 Quantitative Health Sciences, Cleveland, OH, United States

**Keywords:** mindfulness, mindfulness-based cognitive therapy, migraine, mood, depression

## Abstract

**Background:**

Migraine ranks among the leading causes of disability worldwide. Comorbid depressive symptoms are highly prevalent in patients with migraine and are associated with worsened pain severity, greater migraine-related disability, and poorer migraine prognosis. Despite this burdensome comorbidity, the treatment of these co-occurring disorders has rarely been studied. While mindfulness-based cognitive therapy (MBCT) shows promise for addressing both migraine-related disability and depressive symptoms, its traditional format—8 weekly 2-hour sessions in person—creates substantial access barriers, particularly for patients who experience frequent debilitating migraine and mood symptoms.

**Objective:**

The objective of the Treatment for Migraine and Mood (TEAM-M) trial is to evaluate the feasibility of an abbreviated MBCT intervention (MBCT-Brief) delivered via telephone or videoconferencing in adults with migraine and elevated depressive symptoms.

**Methods:**

TEAM-M is a 3-site trial with a goal sample size of 145 adults with episodic migraine and elevated depressive symptoms randomized to MBCT-Brief telephone, MBCT-Brief videoconferencing, or enhanced usual care (EUC). To be eligible, participants must meet the criteria for migraine as defined by the International Classification of Headache Disorders 3rd edition, have ≥1 year of migraine history, and have mild to moderate depressive symptoms (scores 5‐19 on the Patient Health Questionnaire-9).

**Results:**

Our primary outcomes include treatment feasibility, acceptability, and fidelity. Our secondary outcomes include headache disability, migraine-specific quality of life, and depressive symptoms. This trial was funded in May 2021. We began recruitment in November 2023 and completed enrollment in January 2026. As of May 2026, we have randomized 145 participants, of which 104 have completed the intervention and provided data for our primary outcomes. Data analysis is currently in progress, and primary outcome results are expected to be submitted for publication in spring 2027.

**Conclusions:**

The TEAM-M trial addresses a gap in clinical care by evaluating an abbreviated version of MBCT that has scalability and accessibility advantages over full-length MBCT and the potential to address both migraine and depressive symptoms. By reducing the time commitment while maintaining the core MBCT components, MBCT-Brief can potentially address significant access barriers that often prevent patients from receiving evidence-based health care for comorbid physical and mental health symptoms. The remote delivery model offers enhanced scalability. This trial will also yield information about potential differences in telephone versus videoconferencing delivery, which will inform optimal integration into existing primary care and mental health clinic workflows, allowing for improved access to specialized care for mental health and migraine across diverse health care settings.

## Introduction

### Background

Migraine is a highly prevalent disease that affects an estimated 40 million adults in the United States [1,2]. People with migraine experience recurrent episodes of moderate to severe head pain that is associated with nausea and/or vomiting and sensitivity to light and sound in various combinations [3]. Migraine attacks are most common during the peak productive years (eg, ages 20‐40 years) [1,4] and commonly interfere with social and occupational functioning.

Notably, in clinic-based samples, approximately 60% of individuals with migraine report elevated depressive symptoms [5]. Because of their high prevalence and chronic nature, both migraine and depression are leading causes of disability worldwide [6,7]. When comorbid, together they account for a large portion of years lived with disability and have a bidirectional relationship [8,9], with each condition increasing the risk and severity of the other [10]. Compared to those with only migraine, individuals with migraine and depressive symptoms experience greater migraine-related disability (eg, missed work and/or social activities), greater impairment in quality of life, increased use of potentially harmful opioid therapies, twice the total annual medical costs, lower quality of life, and poorer migraine prognosis [11-15]. Despite the significant emotional, social, and economic burden of this comorbidity, few studies have evaluated the management of patients with both migraine and depression. Existing trials suggest that behavioral treatments such as cognitive behavioral therapy show promise in treating both migraine and depressive symptoms [16].

Mindfulness-based interventions have demonstrated efficacy in reducing depressive symptoms, with emerging evidence also suggesting benefits for reducing migraine disability [17], highlighting their strong potential to address this unmet clinical need. Among these approaches, mindfulness-based cognitive therapy (MBCT), which combines mindfulness and cognitive behavioral therapy approaches, has particularly strong evidence for reducing depressive symptoms [18,19] and depression relapse [20,21] and emerging evidence for reducing migraine disability [17,22]. MBCT teaches individuals to focus on present moment experiences through nonjudgmental observations of thoughts and bodily sensations [23], including migraine-related pain and other uncomfortable sensory experiences, while incorporating targeted cognitive techniques designed to address depression and its underlying mechanisms (eg, rumination).

The standard MBCT program follows a structured 8-week format that includes weekly in-person sessions lasting 2 to 2.5 hours each, a full-day meditation retreat, and 40 to 50 minutes of daily meditation practice at home. This intensive protocol is rarely feasible for full participation and engagement among patients with migraine who experience unpredictable disabling symptoms. Both the time commitment and in-person demand can be barriers and particularly burdensome for individuals with chronic health conditions such as migraine, who must balance their medical symptoms and personal and occupational responsibilities. The unpredictability of migraine attacks that involve disabling symptoms of pain, nausea, and vomiting and light or sound sensitivity further creates challenges for in-person attendance. The burden can be especially challenging for young women of reproductive age, who are disproportionately affected by migraine and often navigate multiple familial and professional roles [2]. Our prior research with patients with migraine demonstrated that MBCT delivered through individual hour-long sessions reduced headache disability compared to treatment as usual [17,24], and participants were still often unable to attend treatment appointments in person. In fact, most participants received at least one treatment session over the phone. Thus, a critical need exists for accessible, remotely delivered, and time-efficient interventions for individuals with migraine and depressive symptoms.

We created an abbreviated version of MBCT, MBCT-Brief, that reduces the weekly group session time by half or more (from 120‐150 min to 60 min) thereby reducing participant burden while maintaining alignment with reimbursement for group-based psychotherapy. MBCT-Brief has shown promising outcomes across various delivery formats (telephone and video conferencing) and populations, including reductions in depressive symptoms among individuals with depression [25,26] and decreased stress among women recovering from myocardial infarction [27]. In our prior open-arm pilot study, a remotely delivered MBCT-Brief adapted for individuals with migraine and co-occurring depressive symptoms was feasible, acceptable, and associated with improvements in both headache-related disability and depression severity [28]. While these preliminary findings are promising, they were derived from a small, single-site sample.

In this paper, we describe the protocol for a larger, multisite feasibility and acceptability trial of MBCT-Brief adapted for individuals with migraine and depressive symptoms. To increase generalizability, this study recruited from 3 geographic regions in the United States. This multisite trial will allow us to refine recruitment and retention approaches as well as compare alternative methods of intervention delivery, providing a firm foundation for future trials designed to test the efficacy of remotely delivered MBCT-Brief in individuals with co-occurring migraine and depressive symptoms.

### Aims

The Treatment for Migraine and Mood (TEAM-M) 3-arm trial evaluates the feasibility and acceptability of 2 abbreviated versions of MBCT (MBCT-Brief), one delivered remotely via telephone and the other delivered remotely via videoconferencing. The third arm receives enhanced usual care (EUC), as described below. Our objectives are (1) to establish the feasibility and acceptability of both remote delivery formats and EUC across sites; (2) to demonstrate feasibility of study procedures, including recruitment and retention methods; and (3) to assess fidelity to MBCT-Brief protocols across 3 sites. A key goal is to determine which remotely delivered version of MBCT-Brief demonstrates greater feasibility and acceptability to inform phase III trial design and future clinical implementation. We are also gathering data on patient-centered outcomes including migraine-related disability and depressive symptom severity.

## Methods

### Study Design

This 3-arm, multisite randomized controlled feasibility trial is evaluating an abbreviated MBCT protocol (MBCT-Brief) adapted for individuals with migraine and depressive symptoms and delivered remotely via telephone or videoconferencing compared to EUC.

### Ethical Considerations

The study was approved by the WCG Institutional Review Board (IRB; #20225608). Initial approval was granted on November 15, 2022. The IRBs at all 3 sites—Cleveland Clinic (#23‐584), Einstein College of Medicine (#2022‐13848), and Wake Forest University (#00080688)—rely on WCG IRB as the IRB of record. All participants provide informed written consent prior to participation.

### Preliminary Pilot Work in Preparation for This Trial

We previously adapted the MBCT-Brief protocol for migraine populations and pilot-tested it in adults (N=16) with co-occurring migraine and depressive symptoms randomized to either MBCT-Brief telephone or MBCT-Brief videoconferencing conducted across 2 cohorts (n=8 per cohort) [28]. This preliminary study assessed the feasibility and acceptability of the intervention delivered via the 2 delivery formats (telephone or videoconferencing) in preparation for this trial. Results from the pilot trial demonstrated that both delivery methods were feasible and acceptable, with high usability ratings for remotely delivered components and survey procedures. We observed significant reductions in both headache disability and depressive symptoms from pretreatment to posttreatment [28]. These findings, in combination with other prior studies on MBCT-Brief, provide justification for this multisite study [25-27]. The pilot trial was registered on Clinicaltrials.gov (NCT04992494), with additional details available in the published manuscript [28].

### Participants and Recruitment Methods

#### Eligibility Criteria

##### Inclusion Criteria

Eligible participants met all of the following criteria: (1) International Classification of Headache Disorders 3rd Edition (ICHD-3) [29] criteria for migraine using the validated American Migraine Prevalence and Prevention diagnostic module [30]; (2) self-reported 4 to 20 headache days per month, with at least 1 meeting migraine criteria; (3) mild to moderate depressive symptoms (score 5‐19 on the Patient Health Questionnaire-9 (PHQ-9) [31]; (4) age ≥18 years; (5) ability to read and speak in English; (6) capacity to provide informed consent; (7) ≥1 year of migraine history; and (8) availability and agreeability to attend scheduled group sessions.

##### Exclusion Criteria

We excluded participants if they met any of the following criteria: (1) ICHD-3 criteria [29] for persistent headache attributed to traumatic injury to the head (posttraumatic headache) in the American Migraine Prevalence and Prevention diagnostic module; (2) changes in antidepressant medication within 6 weeks of baseline assessment; (3) starting or changing a migraine preventative treatment (ie, preventive medication, injection, or neuromodulatory device) within 3 months of baseline assessment; (4) changes in acute migraine treatment (ie, acute medication, nasal spray, injection, or neuromodulatory device) started within 4 weeks of enrollment; (5) comorbid psychiatric illness or clinical features that would interfere with participant’s ability to participate in or receive benefit from the intervention, including but not limited to active suicidal ideation, active and elevated trauma-related re-experiencing and dissociation symptoms (as assessed by the Posttraumatic Stress Disorder Checklist for Diagnostic and Statistical Manual of Mental Disorders-Fifth Edition [PCL-5]) [32], active mania, borderline and antisocial personality disorder, cognitive impairment, sensory disabilities, hearing loss, discomfort participating in a group-based intervention, and current substance use disorder; (6) a history of engaging in formal mindfulness-based interventions including mindfulness-based stress reduction, MBCT, acceptance and commitment therapy, and dialectical behavior therapy; (7) engagement in self-reported daily meditation practice at the time of enrollment; (8) lack of willingness to maintain stable current acute or preventive medication dosages for the study duration; (9) any condition that would prevent the participant from being a suitable candidate or interfere with medical care needs; (10) overuse of acute migraine medication that could be associated with medication overuse headache as defined by the ICHD-3 [29].

### Recruitment Methods

We used three primary recruitment methods:

1. Electronic health records: the study team identified patients with an International Classification of Diseases (ICD)-10 diagnosis of migraine seen in neurology and/or primary care clinics within the last 12 months. Patients with an ICD-10 diagnosis of posttraumatic headache were excluded from the list. Of note, depression was not included in the electronic health record data pull criteria.
2. Clinician referral: neurologists at each site identified potentially eligible participants during office visits. Interested patients provided permission for their provider to contact the study team, who then sent the prescreening survey directly to the individual.
3. Social media: paid Facebook (Meta Platforms, Inc) and Instagram (Meta Platforms, Inc) advertisements targeted specific audiences around each study site using age, gender, and zip code.

### Initial Contact, Screening, and Informed Consent

Interested individuals completed a Research Electronic Data Capture (REDCap; Vanderbilt University) prescreening survey with an initial e-consent. Those who preliminarily met the eligibility criteria were scheduled for a phone screening session with a member of the study team, during which the team confirmed final eligibility and obtained full study consent.

### Data Collection

#### Overview

Participants are involved in the study for a total of 9 to 10 months (months are conceptualized as 4 weeks throughout). Participation includes consent provision and screening procedures (~2 weeks) followed by 28 days (4 weeks) of daily diaries completed via REDCap, which monitor headache frequency and migraine symptoms as well as impact on daily activities. The 4 weeks of daily diaries were initially used as a run-in period to determine eligibility prior to randomization. However, at month 13 of the study after 2 cohorts (n=34), this eligibility requirement was dropped due to higher-than-anticipated attrition. The 4-week daily diary data were instead captured post randomization and included to assess the feasibility of daily diary completion and to maintain alignment with standard practices of daily diary assessments in migraine intervention research. Links to daily REDCap surveys were emailed weekly to participants.

**Figure 1.**
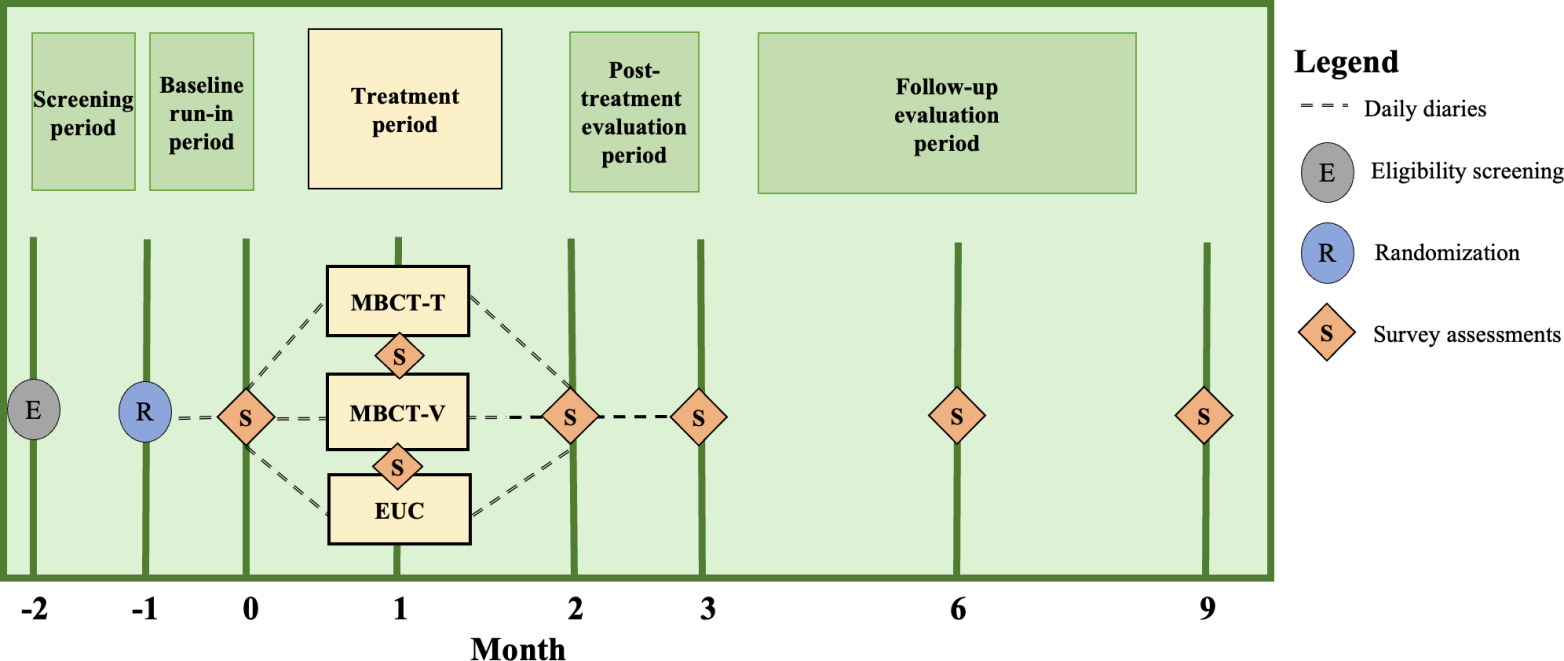
Overview of study procedures. EUC: enhanced usual care; MBCT-Brief-T: mindfulness-based cognitive therapy-brief delivered via telephone; MBCT-Brief-V: mindfulness-based cognitive therapy-brief delivered via videoconferencing.

Following the 4 weeks of daily diaries, all participants (in all study arms) complete a series of online survey assessments at the following time points, defined by the month (M) of participation: baseline (M0); midintervention at week 4 (M1); post intervention (M2); and follow-up at 3 (M3), 6 (M6), and 9 (M9) months (with months conceptualized as 4 weeks). Daily diaries are collected at 3 time points: at the start of the study for 4 weeks (ie, 1 month), during the 8-week intervention (ie, 2 months), and at the 4-week posttreatment evaluation (ie, 1 month; see Figure 1). The diaries at these time points are identical and monitor headache frequency, pain severity, and impact on daily activities.

#### Enrollment and Randomization

When consent was obtained from an entire cohort (n≈30), all participants received a brief video (<10 minutes) via REDCap outlining the study timeline and requirements. Participants communicated their continued interest within 1 week of receiving the video. Only those confirming interest were enrolled and randomized to 1 of the 3 study arms: MBCT-Brief (telephone), MBCT-Brief (videoconferencing), or EUC. Randomization was stratified by sex (male and female) using a randomization list generated by the study statistician with random block sizes of 3 and 6. Participants were blinded to study hypotheses and informed that they would be randomized to one of three 8-week interventions: a skills-based psychological intervention, delivered via telephone or videoconferencing, providing cognitive and mindfulness skills to help manage migraine and cope with stress, negative emotions, and low mood (eg, depression) or an online education program focused on the diagnosis and treatment of migraine and depression.

#### Self-Report Measures of Headache, Mood, and Other Psychological Constructs

Validated self-report measures of headache symptoms, mindfulness, and psychological variables are administered via REDCap at baseline (M0); midintervention (M1); post intervention (M2); and follow-up at 3 (M3), 6 (M6), and 9 (M9) months. Multimedia Appendix 1 provides an overview of all measures.

### Study Arms

#### MBCT-Brief for Migraine

In this study, we used a previously abbreviated version of MBCT (MBCT-Brief) that we modified for individuals with migraine (see Table 1).

**Table 1.** Overview of the abbreviated version of mindfulness-based cognitive therapy (MBCT-Brief) adapted for individuals with migraine and comorbid depressive symptoms.

Session	Theme	Aims	Agenda: content (duration in minutes)
Week 1	Awareness and automatic pilot	Recognize the difference between conscious awareness and automaticity (“doing mode” vs “being mode”) Cultivate direct experience via the senses Recognize when direct experience is interrupted by thinking Develop awareness of how automatic pilot contributes to distress	Welcome and orientation (10) Participant introductions (5) Practice: mindful eating (10) Inquiry (15) Homework planning (10) Closing meditation (5)
Week 2	Awareness of the body	Cultivate direct experience of the body (sensing vs thinking) Develop ability to place and shift attention Develop awareness of emotion Begin to develop awareness of aversion and attachment	Practice: body scan (20) Inquiry (15) Migraine warning signs (5) Homework review (10) Homework planning (5)
Week 3	Gathering the scattered mind	Develop attention to salient sensations of the breath and in the body Cultivate patience, curiosity, and compassion	Practice: mindfulness of breath and body (20) Inquiry (15) Homework review (15) Homework planning (5)
Week 4	Recognizing thinking	Recognize thoughts and thinking Develop awareness of reactivity (attachment or aversion) to pleasant and unpleasant thoughts Develop awareness of the passing nature of thoughts and thinking	Practice: mindfulness of thoughts and thinking (20) Inquiry (15) Homework review (15) Homework planning (5)
Week 5	Thoughts are not facts	Recognize the link between thoughts/interpretations, sensations, emotions, and urges/behavior Identify personal overlearned reactions (ie, habitual thoughts and thought patterns as well as ways of behaving/acting) that perpetuate distress, depression, and anxiety Recognize that there are signature thoughts for depression and anxiety that are universal and that perpetuate symptoms	Thoughts and feelings exercise (5) Inquiry (15) Depression and anxiety symptom review (10) Three-step breathing space (10) Homework review (15) Homework planning (5)
Week 6	Allowing or letting be	Develop ability to “be with” the unwanted (improve the ability to respond rather than react in the face of difficulty)	Practice: working with difficulty (20) Inquiry (15) Homework review (15) Homework planning (5)
Week 7	Taking care of myself	Recognize the bidirectional link between activity and mood Recognize that choices can be made about what activities to engage in and how (ie, changing attitude and recognizing habitual unhelpful thinking patterns) to support mood, recovery, and coping	Practice: breath + difficulty (10) Daily activities exercise (30) Homework review (15) Homework planning (5)
Week 8	Maintenance	Identify how mindfulness practices have supported coping with migraine and depression Identify what continued practice will look like and plan for what might get in the way	Practice: body scan (20) Inquiry (5) Homework review (10) Lessons learned (15) Handout review (5) Concluding meditation (5)

##### MBCT-Brief

MBCT-Brief was developed prior to this trial by coauthor PV, a certified MBCT facilitator, in collaboration with coauthor AJS and colleagues. MBCT-Brief has been previously validated in patients with depression [25]. The abbreviated curriculum maintains the fundamental structure and pedagogical principles of the full-length MBCT intervention while also reducing the session time to 1 hour per week. Each session mirrors the standard MBCT format: guided mindfulness practice and inquiry, at-home practice review, and assignment and review of new at-home practice for the next week. The sequencing of session themes, their underlying rationale, and core learning objectives remains largely unchanged. Critically, MBCT’s experiential and reflective therapeutic approach has been preserved.

The key modifications included reducing the duration of the weekly sessions from 2 hours to 1 hour, reducing the duration of the at-home daily mindfulness practice from 45 to 60 minutes to 10 to 20 minutes, and eliminating the full-day silent retreat. Session time was decreased by reducing the number of mindfulness and cognitive activities, the duration of meditation practice (reduced to a maximum of 20 minutes), and the time dedicated to facilitator-guided inquiry (reduced to a maximum of 15 minutes). Decisions to retain or remove activities from the standard MBCT protocol were guided by the three overarching aims outlined by Segal et al [33] (p. 83): (1) increasing moment-to-moment awareness of internal experiences (eg, sensations, emotions, and impulses), (2) developing alternative ways of relating to these experiences, and (3) learning to choose skillful responses to unpleasant internal experiences. The team retained a carefully selected subset of exercises that introduced and built upon these core skills (Table 2).

**Table 2.** Core exercises retained from the full-length mindfulness-based cognitive therapy (MBCT) protocol and rationale.

Meditative practice	Rationale
Raisin exercise	This practice reveals the experience of automatic pilot as contrasted with the experience of focused awareness, the basis for building mindfulness and acceptance skills.
Body scan	Developing bodily and sensory awareness is a foundational skill and requirement for the later work of tolerating discomfort and distress and building acceptance skills.
Mindfulness of breath and body	This practice expands on bodily and sensory awareness skills for developing focus and concentration, which are key to attention regulation and thus, later, emotion/distress regulation and interrupting rumination, etc.
Mindfulness of thoughts and thinking	This practice is a critical prerequisite to working with difficulty exercise (below) because of the focused training that it provides in recognizing thoughts and the thinking process and seeing them as distinct from emotions and sensations.
Working with difficulty	This practice is viewed as the capstone practice because it brings together all the skills learned in earlier meditations and sessions, offering participants a radically new approach to coping.
Three-step breathing space	This brief practice is designed to provide a quick, structured pause to facilitate shifting from automatic pilot to mindful awareness acting as a mini-meditation reset throughout the day.
Three-step breathing space—responsive (and with action)	This brief practice is designed to function as a quick and accessible tool to be used in response to life’s challenges.
Other exercises	
Thoughts and feelings exercise (walking down the street)	This exercise illustrates the connection between thoughts and feelings, a key tenet of cognitive behavioral therapy and MBCT, and that our interpretations of events influence our behavior and mood.
Unpleasant experiences calendar	This exercise helps to identify unpleasant feelings and the reactions to these feelings, revealing a tendency to avoid what we do not like.
Depression and anxiety symptom review	This exercise helps to identify symptoms and cues marking a shift in mood state that are not personal.
Nourishing/depleting activities	This exercise illustrates the link between behavior and mood and reveals opportunities to mindfully and intentionally modify daily routines to promote positive mood.
Action plan	This exercise summarizes individual triggers identified over the course of the program and outlines a skillful plan of response to mitigate risk for relapse.

##### Adaptation for Migraine

The original full-length MBCT protocol was previously adapted for migraine by coauthor ES and colleagues [34] and demonstrated reductions in migraine-related disability [17]. Adaptations included migraine-specific education and treatment rationale, as well as exercises designed to enhance awareness of migraine-related symptoms, sensations, and behavioral reactions. These adaptations were incorporated by coauthor PV into the MBCT-Brief curriculum for individuals with migraine. Table 1 provides a detailed session-by-session overview of this curriculum.

##### Materials and Home Practice

Prior to the first intervention session, participants in both MBCT-Brief arms received a printed and electronic version of the participant session-by-session workbook containing psychoeducational material about mindfulness, depression, migraine, and core MBCT concepts, along with home practice instructions and tips, tracking logs, and assigned worksheets and readings. While full-length MBCT prescribes roughly 45 minutes of daily at-home mindfulness practice, MBCT-Brief reduces this to 20 minutes of mindfulness practice plus worksheets and readings, totaling approximately 30 minutes daily. The home practice schedule mirrors the full-length protocol, using shortened audio recordings for mindfulness practices. Participants randomized to MBCT-Brief record observations (eg, sensations, thoughts, and feelings), reflections, and/or the duration of home practice in a log provided in the intervention workbook.

##### Delivered via Telephone or Videoconferencing

All sessions are conducted and recorded using Cisco Webex, with the video function deactivated for the MBCT-Brief telephone group. Groups include an average of 8 participants who are expected to attend all 8 sessions and complete the daily home practice. If a participant cannot attend a session, they are encouraged to complete a make-up session to access the respective group recording (audio or video, depending on group assignment) via a secure link. The recording link is accessible only during the scheduled make-up time. After completing the session, participants receive a REDCap survey asking about the length of the recording watched and perceived helpfulness of the session exercises. If a participant requests more than 2 make-up sessions, the study team contacts them to identify and collaboratively address barriers to live participation.

##### Facilitators

All MBCT-Brief sessions are facilitated by licensed mental health clinicians or masters-level trainees practicing under supervision. Facilitators have education and training in clinical psychology or related fields and in cognitive and behavioral theories for depression and anxiety. All facilitators are experienced in guiding group meditations and have completed foundational training in either MBCT or mindfulness-based stress reduction. Clinicians and masters-level graduate students completed 35 hours of specialized MBCT-Brief protocol training provided by PV. This training included two integrated components: (1) didactic training on core cognitive behavioral therapy and mindfulness principles and concepts, MBCT-Brief program structure and learning objectives, orientation meeting procedures, basics of group facilitation, and session time management strategies and (2) experiential training on guiding meditations and conducting inquiry. Training intensity and focus areas were tailored to each facilitator’s baseline knowledge and mindfulness experience, as determined by PV’s assessment of professional competencies. Facilitators were also trained by headache specialists, REW and ES, on migraine disease and its management. All facilitator training was conducted virtually using videoconferencing.

During intervention delivery, facilitators attend weekly supervision with PV. PV reviews session recordings and uses the Mindfulness-Based Interventions: Teaching Assessment Criteria (MBI:TAC) and the Mindfulness-Based Teaching and Learning Companion (MBI:TLC)—widely used tools for developing and ensuring facilitator competence [35,36]—to support facilitator skill development and adherence to the curriculum.

### Enhanced Usual Care

Participants randomized to the EUC condition receive educational information for migraine management and depression risk reduction, along with referrals to mental health and migraine resources. This information is delivered through 8 weekly online education modules based on content from education control groups used in previous behavioral migraine management studies (NCT03706794 and NCT03982316), adapted to include information on depressive symptoms, including vignettes depicting people with migraine and depressive symptoms to demonstrate key concepts. The modules are released weekly to mirror the MBCT-Brief schedule and cover topics including the relationship between migraines and depressive symptoms, treatment options for these co-occurring conditions, lifestyle factors for migraine and depressive symptoms, and communicating with health care providers (see Table 3). Notably, the EUC materials do not include any mindfulness-based content or practices. Participants engage with the education modules individually rather than in groups, and engagement is tracked electronically by the secure hosting website. Education modules are a pragmatic control and are not attention matched. They take approximately 15 minutes to complete weekly. When EUC participants are notified of their group assignment, they are told that they will be receiving 8 weekly self-management strategies curated by mental health and migraine specialists to help manage mood and migraine.

**Table 3.** Content and structure of the control condition (enhanced usual care).

Week	Module	Description
1	What is migraine?	Diagnosis and etiology of migraine as well as the role of the physician
2	Living with migraine	Prevalence of migraine, impact of migraine symptoms on activities of daily living, and the social stigma associated with migraine
3	Migraine management	Strategies for managing migraine, including acute and preventive treatment
4	Mood and migraine	The relationship between depressive symptoms and migraine and strategies for mood management
5	Stress and migraine	Stress as a common migraine trigger and strategies for stress management
6	Consistent living	Maintenance of a consistent eating and hydration routine to prevent and manage migraine
7	Self-care	Prioritization of health and mood, making time for “nourishing” activities, and overcoming self-doubt
8	Next steps	A summary of the previous modules and how to set specific, measurable, achievable, relevant, and time-bound (SMART) goals

### Treatment Adherence and Fidelity

Among participants randomized to MBCT-Brief, we measure treatment adherence by tracking session attendance and homework compliance with the at-home daily practice logs provided in the workbook. For the EUC condition, REDCap automatically tracks the number of education modules that each participant completes. We also take steps to increase the likelihood that participants adhere to treatment. First, we use motivational interviewing strategies to assess participants’ interest in the study as well as their confidence in being able to attend most of the 8 intervention sessions on a scale of 1 to 10 (1, not interested/no confidence; 10, very interested/very confident) during screening. If a potential participant reports low interest in the study (score of ≤5), a study coordinator contacts that individual using motivational interviewing techniques to evaluate and enhance motivation for continuing through the study screening and enrollment process. If a potential participant continues to report low confidence in attending the intervention sessions (score of ≤5), they are advised to consider waiting to join the study until a more suitable time for their schedule. Second, the importance of session attendance is stated during the orientation call. Third, the study coordinator sends email reminders about the date and time of each session to every participant each week and assists with scheduling a make-up session if a participant needs to miss a live session. Lastly, during each weekly group session, the facilitator reviews the at-home practice assignments with participants to identify and overcome barriers to completion.

We manage treatment fidelity through the use of a facilitator treatment manual, session fidelity checklists that are completed by the facilitator, weekly supervision, and independent raters.

### Participant Safety Monitoring

The trial is conducted in compliance with the current Good Clinical Practice guideline [37] and the principles of the Declaration of Helsinki [38]. Individuals are not eligible to enroll in the study if they are at high risk of an adverse event throughout the intervention period or require more intensive, focused treatment. This includes individuals with active suicidal ideation, psychosis, or significantly elevated trauma-related dissociative symptoms that would benefit from specialized trauma-focused interventions before participating in a mindfulness-based approach. This also includes people with medication-taking patterns that indicate potential medication overuse–induced headache. These exclusions reflect clinical considerations for participant safety and optimal treatment sequencing rather than contraindications to MBCT. Individuals meeting these criteria are referred to appropriate psychiatric or neurological services and connected with crisis or emergency services for acute needs, as needed.

The study team regularly monitors the trial for adverse events, both on weekly questionnaires and during intervention sessions. The weekly questionnaires assess new or worsening symptoms of PTSD, dissociation, pain, migraine, and any other troubling medical experiences. Each adverse event is discussed at weekly team meetings, during which the study team formulates a personalized plan for the at-risk participant to address the event and to identify any potential emerging patterns of adverse events. Considering that this trial includes individuals with elevated depressive symptoms, safety monitoring entails close observation of clinically significant increases in depressive symptoms and reports of suicidal ideation. If a participant reports suicidal ideation on the depression measure (administered at baseline and months 1, 2, 3, 6 and 9), weekly adverse event surveys, or verbally during intervention sessions, the study team implements the suicide safety protocol. As part of this protocol, the Columbia-Suicide Severity Rating Scale (C-SSRS) is either administered automatically through REDCap if suicidal ideation is endorsed on depression measures or adverse event surveys or administered verbally over the phone if suicidal ideation is reported during intervention sessions. The study team then addresses the participant’s suicidal ideation according to the corresponding risk level from the C-SSRS, with interventions including providing local mental health resources and performing warm handoffs with the 988 Suicide and Crisis Line.

All study personnel received training in suicidal ideation monitoring and safety protocol implementation, with individuals who interact directly with participants completing specialized suicide assessment and prevention training sponsored by the Suicide Prevention Resource Center [39]. This training focuses on reducing access to lethal means and working with individuals at risk for suicide and their families to minimize risk factors. Licensed clinical psychologists and neurologists at each site are available to provide oversight for psychiatric adverse events and ensure appropriate clinical response to safety concerns.

### Statistical Methods

#### Primary Outcomes

##### Treatment Fidelity

Treatment fidelity is evaluated by 2 or more independent raters with training in and familiarity with the MBCT-Brief protocol. Raters review and score audio recordings of all MBCT-Brief sessions (both telephone and videoconferencing) using the adherence component of the MBCT-Teaching Assessment Criteria (MBCT-TAC), which was adapted from the MBCT Adherence Scale (MBCT-AS), a standardized measure of fidelity [40]. Raters demonstrated competency in using the MBCT-TAC through standardized training procedures to ensure unbiased assessment with a target of 85% interrater reliability. Adherence is rated for inclusion of formal components such as homework assignment, guided exercises, and inquiry as well as teaching goals (eg, learning to place and hold attention and recognizing aversion) for each session on a 0 to 2 scale: 0 (“no evidence”), 1 (“slight evidence”), and 2 (“definite evidence”). Each MBCT-Brief treatment arm will be considered as administered with fidelity if the mean MBCT-TAC adherence scores are ≥2.5.

##### Treatment Feasibiliy

Treatment feasibility is defined as achieving an average of 75% completion of assigned sessions or modules and is assessed by monitoring attendance logs. A threshold of 75% session attendance is well established in the current literature and aligns with best practices for scalable and sustainable behavioral health interventions [41,42]. For the MBCT-Brief arms, a study coordinator present at each intervention session tracks the number of treatment sessions attended. For the EUC arm, participants self-report each week if they completed the weekly module, and REDCap automatically tracks the number of education modules that each participant completes.

##### Treatment Acceptability

Acceptability, defined as treatment satisfaction, is assessed post intervention at 8 weeks using the self-report Client Satisfaction Questionnaire (CSQ-8) [43]. The CSQ-8 is an 8-item self-report measure commonly used in behavioral treatment trials to evaluate satisfaction with mental health services. Items are rated on a 4-point scale, yielding a total score ranging from 8 to 32. The measure has consistently demonstrated strong reliability and validity. In this study, a total CSQ-8 score greater than 24 is indicative of acceptable treatment satisfaction.

### Secondary Outcomes

#### Headache Disability

Headache disability is assessed at M0, M1, M2, M3, M6, and M9 using the Headache Disability Inventory (HDI) [44]. The HDI is a 25-item self-report survey which assesses perceived emotional and functional impact of headache on daily activities. A sample item is “Because of my headaches I feel restricted in performing my routine daily activities,” with the response options of “Yes,” “Sometimes,” and “No,” assigned 4, 2, and 0 points, respectively. A total percentage score (maximum 100) is calculated to gauge disability: 10 to 28 (mild), 30 to 48 (moderate), 50 to 68 (severe), and 72+ (complete), with higher scores representing greater disability.

#### Migraine-Specific Quality of Life

Migraine-specific quality of life is assessed at M0, M1, M2, M3, M6, and M9 using the Migraine-Specific Quality of Life Questionnaire (MSQ; version 2.1) [45]. The MSQ is a 14-item self-report survey that assesses quality of life over the past 4 weeks in people who experience migraine. Items comprise 3 subscales: role restriction, role prevention, and emotion function. Each item is scored on a Likert scale from 1 (“None of the time”) to 6 (“All of the time”). Total scores range from 14 to 84. The change in migraine-specific quality of life is evaluated using the slope of change from month 0 to month 3.

#### Depressive Symptoms

Depressive symptoms are assessed at M0, M1, M2, M3, M6, and M9 using the Patient-Reported Measurement Information System-Depression (PROMIS-D) and Quick Inventory of Depressive Symptomatology-Self-Report 16 (QIDS-SR16), both of which evaluate symptoms over the past 7 days [46]. The PROMIS-D includes 8 items that are rated on a scale from 1 to 5, with higher scores reflecting higher symptom severity. Total scores are converted to T scores based on normative data. The QIDS-SR 16 includes 16 items rated on a scale from 0 to 3, yielding a total score ranging from 0 to 27. Score ranges are categorized as follows: 1 to 5 (no depression), 6 to 10 (mild), 11 to 15 (moderate), 16 to 20 (severe), and 17 to 27 (very severe). Changes in depressive symptoms across both measures are evaluated using the slope of change from baseline (month 0) to month 3.

### Planned Analyses

#### Descriptive Statistics

Basic descriptive statistics (eg, means and SDs, frequencies and percentages, or medians and IQRs) for variables of interest will be computed. Distributions of each variable will be examined. Graphical methods will be used to further explore the data when appropriate. Differences in demographics and clinical characteristics at baseline will be evaluated across the 3 treatment arms. Any significant differences on demographic variables between treatment groups and all stratification variables will be included as covariates in secondary adjusted models for the secondary analyses described below.

#### Primary Analyses

As this is a randomized trial, primary analyses will be unadjusted for baseline covariates. Adjusting for sex will also be considered in additional analyses. Descriptive statistics (mean and SD) will be calculated for the MBCT-TAC in total and across each treatment arm (MBCT-Brief telephone, session n=48 vs MBCT-Brief videoconferencing, session n=48). Treatment session adherence will be summed to indicate the number of treatment sessions attended and descriptive statistics (mean and SD) will be calculated. A treatment arm will be considered feasible if, on average, participants attended 75% of sessions across all sites. Descriptive statistics (mean and SD) will be calculated for the CSQ-8 scores across each treatment arm (MBCT-Brief telephone, MBCT-Brief videoconferencing, and EUC). A treatment arm will be considered acceptable if CSQ-8 scores are>24 across all sites.

We will explore whether treatment fidelity, treatment feasibility, or treatment acceptability differ significantly between MBCT-Brief telephone and MBCT-Brief videoconferencing. All analyses will use a 2-tailed α set at .05. To evaluate differences in treatment fidelity, we will use a multilevel linear mixed effects model to evaluate differences between MBCT-Brief telephone and MBCT-Brief videoconferencing on the MBCT-TAC (72 sessions nested in 6 treatment groups). The treatment site will be included as a covariate, and repeated measures will be taken into account using random effects. To evaluate differences in feasibility, we will use a multilevel linear mixed effects model using random effects to evaluate differences between MBCT-Brief telephone and MBCT-Brief videoconferencing in treatment session adherence (96 participants nested in 6 treatment groups). To evaluate differences in treatment acceptability using scores from the CSQ-8, we will use cross-sectional analysis.

#### Secondary Analyses

Secondary analyses will include adjusted models controlling for baseline demographic and clinical characteristics that differ significantly between treatment arms. We will evaluate the change in headache disability, migraine-specific quality of life, and depressive symptoms by calculating the slope of change from month 0 to month 3. Multilevel mixed effects models will evaluate changes in outcomes at the month level across treatment arms (MBCT-Brief telephone vs MBCT-Brief videoconferencing vs EUC). Within-person repeated measures (monthly) will be considered by using person-level random effects.

### Missing Data

No missing data are anticipated for the primary outcomes related to treatment fidelity feasibility. Efforts are made to obtain CSQ-8 scores from all participants; however, missing CSQ-8 data will be singly imputed as the lowest possible score on the CSQ-8, indicating dissatisfaction with the treatment, and if a participant drops out of the study prior to month 2 and cannot be reached, a total score of 8 on the CSQ-8 will be imputed, reflecting dissatisfaction with all treatment domains. For secondary outcomes, missing data will be estimated using restricted maximum likelihood within mixed models procedures. The best-fitting covariance structure for each outcome will be selected using the Akaike Information Criterion. Mixed models allow for unbiased parameter estimations, even when data are not missing at random [47].

### Data Management

Data are collected by trained research assistants and study coordinators using electronic questionnaires administered via REDCap, a secure web-based platform for managing online surveys and databases. All surveys are deidentified and coded with unique subject identifiers to maintain participant confidentiality. Initial screening data are collected and stored on each participating site’s local REDCap platform, with only the project manager having access to data from all 3 sites for monitoring purposes. Following randomization, participants are transferred to the main REDCap database hosted at Cleveland Clinic, which serves as the central database for all intervention-related data collection and surveys. The Cleveland Clinic study team is responsible for sending and monitoring all postrandomization data collection while maintaining the site coordinators’ access to monitor and address adverse events as needed.

To ensure data quality, every 2 weeks, the study team members conduct reviews of all REDCap data collection forms to identify missing data, discrepancies, or other identified issues. Study staff follow up with participants who have not completed surveys within 1 week of administration. Any discrepancies or data-related issues are reviewed and corrected by the project manager. This comprehensive approach allows for ongoing monitoring of study progress, participant engagement, and the collection of high-quality data.

### Sample Size and Power Estimates

Power calculations were informed by preliminary data (means and SDs) and were adjusted for the 25% dropout rate. For treatment fidelity, all sessions met the benchmark in preliminary testing; within a 95% CI, we will achieve a power above 0.99 to detect a mean fidelity score at or above the benchmark. Similarly, the majority of participants met benchmarks for both feasibility and acceptability in preliminary testing. Thus, within a 95% CI, we will achieve a power above 0.89 to detect mean feasibility and acceptability score at or above the benchmarks [28].

## Results

This study was funded in May 2021. Participant recruitment began in November 2023, and enrollment was completed in January 2026. As of May 2026, we prescreened 1874 individuals, phone-screened 317 individuals, and randomized 145 participants, and 104 participants completed the intervention and provided data for our primary outcomes. Data analysis is currently in progress,and primary outcome results are expected to be submitted for publication in spring 2027.

In our original protocol, the first 4 weeks of daily diaries were used as a run-in period to determine the inclusion criterion with two requirements for enrollment and randomization: (1) a minimum daily diary compliance of ≥25 of 28 entries (89.28% completion rate) and (2) confirmation of migraine diagnosis through documented headache patterns (4‐14 headache days with at least 1 episode meeting the International Classification of Headache Disorders migraine criteria). Following this eligibility criterion, among the first 2 cohorts, 134 participants consented to participate; 89 of these 134 participants (66.42%) were eligible after the phone screening phase, but only 59 of 134 (44.03%) completed the run-in period. The individuals who did not complete the run-in period included those who never initiated the run-in period (14/134, 10.45%) as well as those who began but did not complete the run-in phase (16/134, 11.94%). Of the 59 participants who completed the run-in period, only 34 (34/134, 25.37%) met the eligibility criteria for randomization, prompting the need for reevaluation of study procedures. The study team identified that none of the primary outcomes for the clinical trial relied on headache diary data. Therefore, the baseline headache daily diary log period was transitioned to occur after randomization, removing the inclusion criteria of daily diary adherence and migraine diagnosis via headache log. We retained the 4 weeks of daily diaries at baseline (post randomization) to collect baseline data as well as feasibility and acceptability data on daily diary compliance. Headache frequency and migraine diagnosis were assessed through participant self-report in the prescreening survey, with confirmation during phone screening, an approach shown to be comparable to daily diary reporting for headache frequency assessment [48]. Additionally, we expanded our headache frequency inclusion criteria from 4 to 14 days per month to 4 to 20 days per month to align with relevant guidelines [49] and the landmark clinical trial for behavioral migraine management [50].

## Discussion

### Study Significance and Future Research

The primary objective of the TEAM-M multisite trial is to evaluate the feasibility and acceptability of MBCT-Brief for co-occurring migraine and depressive symptoms. Compared to the full-length intervention, MBCT-Brief reduces the time commitment by more than half, an important modification given that time demands are a known barrier to participation in mindfulness-based interventions for this population [17] and for individuals with chronic health conditions [51,52], as well as more broadly [53-55]. Additionally, the abbreviated format allows clinicians to treat more patients within the same time frame without adding clinical workload, addressing a significant access concern given the shortage of trained MBCT instructors [56]. While this trial does not aim to address the shortage of instructors directly, this abbreviated format aligns with reimbursement policies, offering a more economically sustainable model for providers and health care systems. Our long-term goal is to evaluate the efficacy of MBCT-Brief and to improve its accessibility and scalability for depression care broadly and in the context of comorbid conditions such as migraine. Therefore, our study is an important step in addressing a critical gap in care: the lack of integrated treatments for co-occurring migraine and depressive symptoms.

Another key objective of this trial is to determine which remote delivery method (telephone or videoconferencing) demonstrates greater feasibility and acceptability, which will inform the selection of a single method in a phase III trial. Each delivery method warrants consideration. Telephone delivery offers important advantages beyond accessibility, including reduced barriers for individuals experiencing depression-related symptoms, such as appearance-related concerns or low self-esteem, which may impede participation in video-based care. Additionally, telephone delivery eliminates requirements for an internet connection or video-enabled device. However, video delivery may enhance group cohesion when members can see one another, which may in turn improve outcomes, although this requires further study. Insurance reimbursements are another point of consideration, as coverage for audio-only telehealth services varies significantly by payer, with some coverage (including Medicare) having ended in September 2025. These changes may affect long-term accessibility and scalability for telephone-delivered MBCT.

Feasibility and acceptability randomized controlled trials, like this one, are critical for laying the groundwork for larger efficacy trials by testing and refining the intervention design as well as recruitment, enrollment, and other study procedures. For example, our original protocol that included the 28-day run-in period as an inclusion criterion proved burdensome and revealed significant enrollment challenges. We experienced a higher-than-expected percentage of participants not meeting either the required diary completion threshold, the headache frequency criteria, or both, ultimately hindering study progress. As a result, we modified the protocol to decouple daily diary data collection from the inclusion criterion. We revised the assessment of headache frequency and migraine diagnosis to rely on participant self-report in the prescreening survey, an approach that has been found to be comparable to daily diary reporting for headache frequency assessment [48]. We also expanded the headache frequency inclusion criterion from 4 to 14 days per month to 4 to 20 days per month to account for day-to-day fluctuations in episodic migraine that may increase monthly headache days and to align with other behavioral migraine studies [57,58]. Daily diaries were used to assess the feasibility and acceptability of daily diary compliance to inform future trial design. These protocol modifications significantly improved enrollment rates and strengthened both the current trial and the planned phase III efficacy trial.

### Limitations

We acknowledge several limitations of this randomized controlled trial. While we are collecting preliminary data on headache disability, migraine-specific quality of life, and depressive symptoms to inform power analyses for a future efficacy trial, any reported efficacy data in future publications should be interpreted as exploratory. Additionally, we rely on self-report for collecting data on participants’ at-home practice, which is common in mindfulness-based trials but introduces potential bias. Future studies would benefit from the incorporation of objective tracking methods (eg, app-based monitoring of guided meditation use) to more accurately assess engagement in at-home practice.

## Supplementary material

10.2196/93627 Multimedia Appendix 1 Self-report measures completed by participants at each time point.

10.2196/93627 Peer Review Report by ZAT1 PJ (10) - National Center for Complementary and Integrative Health Special Emphasis Panel, Exploratory Clinical Trials of Mind and Body Interventions (MB) (National Institutes of Health, USA).

## Abbreviations

C-SSRS: Columbia-Suicide Severity Rating Scale
CSQ-8: 8-item Client Satisfaction Questionnaire
EUC: enhanced usual care
HDI: Headache Disability Inventory
IRB: Institutional Review Board
MBCT: mindfulness-based cognitive therapy
MBCT-AS: Mindfulness-Based Cognitive Therapy Adherence Scale
MBCT-Brief: abbreviated version of the mindfulness-based cognitive therapy intervention
MBCT-TAC: Mindfulness-Based Cognitive Therapy-Teaching Assessment Criteria
MBI:TAC: Mindfulness-Based Interventions: Teaching Assessment Criteria
MBI:TLC: Mindfulness-Based Interventions: Teaching and Learning Companion
MSQ: Migraine-Specific Quality of Life Questionnaire
PCL-5: Posttraumatic Stress Disorder Checklist for Diagnostic and Statistical Manual of Mental Disorders-Fifth Edition
PHQ-9: Patient Health Questionnaire-9
PROMIS-D: Patient-Reported Measurement Information System—Depression
QIDS-SR16: Quick Inventory of Depressive Symptomatology-Self-Report 16
REDCap: Research Electronic Data Capture
TEAM-M: Treatment for Migraine and Mood
