# Supplementary material for: Evaluating an Abbreviated Version of Mindfulness-Based Cognitive Therapy Delivered via Telephone or Videoconferencing Compared to Enhanced Usual Care—Treatment for Migraine and Mood (TEAM-M) Study: Protocol for a Three-Arm Multisite Randomized Controlled Feasibility Trial
Source: JMIR Res Protoc. 2026 Jun 5;15:e93627. doi: 10.2196/93627 (PMC13240983; doi:10.2196/93627)
Supplement: Multimedia Appendix 1 [file resprot-v15-e93627-s001.docx]

|  | |  |  |
| --- | --- | --- | --- |
| **Appendix A.** Self-report measures completed by participants at each timepoint. | |  |  |
| **Assessment** | **Construct assessed** | | **Time points** |
| **Screening Measures** |  | |  |
| Health history | Migraine diagnosis, medication usage, psychiatric diagnosis | | Prescreen, M0 |
| Demographic and background information | Age, race, ethnicity, English-speaking, hearing loss, history of mindfulness practice | | Prescreen |
| Interest/Commitment | Confidence in availability and interest in participating in the research study | | Prescreen |
| American Migraine Prevalence and Prevention Study Diagnostic Module | Migraine symptoms | | Phone screen |
| Patient Health Questionnaire (PHQ-9) – Items 1-8 | Depressive symptoms | | Prescreen |
| Patient Health Questionnaire (PHQ-9) – Item 9 | Suicidality | | Phone screen |
| PTSD Checklist for DSM-V (PCL-5) | Posttraumatic stress symptoms | | Prescreen |
| Dissociative Subtype of PTSD Scale (DSPS; Items 1,3,5,7,8,9,12) | Presence of dissociation/depersonalization and derealization symptoms | | Prescreen |
| Drug Abuse Screening Test (DAST-10) | Consequences related to drug abuse | | Prescreen |
| Single Item: 6-Month Mania History | Bipolar disorder (mania) symptoms | | Prescreen |
| McLean Screening Instrument for BPD | Borderline personality disorder | | Prescreen |
| Columbia-Suicide Severity Rating Scale (C-SSRS)† | Suicide risk severity, immediacy, and level of needed support | | Phone screen, M0, Weekly during intervention, M1, M2, M3, M6, M9 |
| **Patient Reported Outcomes** |  | |  |
| *Migraine Measures* |  | |  |
| Headache Disability Inventory (HDI) | Perceived emotional and functional impact of headache on daily activities | | M0, M1, M2, M3, M6, M9 |
| Migraine-Specific Quality of Life Questionnaire (MSQ)+ | Quality of life over the past 4 weeks in people with migraine | | M0, M1, M2, M3, M6, M9 |
| Migraine Disability Assessment (MIDAS)+ | Quantifies headache-related disability | | M0, M1, M2, M3, M6, M9 |
| Headache Management Self-Efficacy Scale (HMSES)+ | Sense of self-efficacy in managing headache disorder | | M0, M2 |
| Pain Catastrophizing Scale (PCS)+ | Tendency to magnify the threat of pain, feel helpless in the presence of pain, and feel unable to prevent and inhibit pain-related thoughts. | | M0, M1, M2, M3, M6, M9 |
| Head Pain Acceptance Questionnaire (HPAQ) | Acceptance of headache pain | | M0, M1, M2, M3, M6, M9 |
| Migraine Interictal Burden Scale (MIBS)+ | Migraine-related burden in 4 domains: work/school, family/social, planning, and emotional functioning | | M0, M1, M2, M3, M6, M9 |
| Allodynia Symptom Checklist (ASC)+ | Frequency of allodynia symptoms | | M0, M2 |
| Stigma Scale for Chronic Illness (SSCI-24)+ | Enacted and internalized migraine-related stigma | | M0, M2 |
| Headache Frequency+ | Headache activity | | Prescreen, phone screen |
| Average Headache Attack Pain Intensity+ | Pain intensity | | Daily diary |
| *Mental Health Measures* |  | |  |
| Quick Inventory of Depressive Symptomatology (QIDS)+ | Depressive symptoms | | M0, M1, M2, M3, M6, M9 |
| Patient-Reported Outcomes Measurement Information System-Depression (PROMIS-D)+ | Depressive symptoms | | M0, M1, M2, M3, M6, M9 |
| Generalized Anxiety Disorder-7 (GAD-7)+ | Anxiety symptoms | | M0, M1, M2, M3, M6, M9 |
| Perceived Stress Scale (PSS-14) | Severity of stressors | | M0, M1, M2, M3, M6, M9 |
| *Other Measures* |  | |  |
| Five-Facet Mindfulness Questionnaire (FFMQ-15)+ | Trait mindfulness | | M0, M1, M2, M3, M6, M9 |
| Patient Reported Outcomes Measurement Information System – Sleep Disturbance (PROMIS-SD)+ | Sleep quality | | M0, M1, M2, M3, M6, M9 |
| Multidimensional Assessment of Interoceptive Awareness (MAIA-2)+ | Dimensions of interoceptive awareness, including body listening, emotional connection, and self-regulation. | | M0, M1, M2, M3, M6, M9 |
| Psychological Sense of Community Scale (PSOC)+ | Participants’ sense of community in the treatment group | | M2 |
| *Feasibility and Acceptability Measures* |  | |  |
| Credibility Expectancy Questionnaire (CEQ) | Treatment credibility and outcome expectancy | | M0 |
| Client Satisfaction Questionnaire (CSQ-8)* | Overall satisfaction with therapeutic services received | | M2 |
| Satisfaction With Therapy and Therapist Scale-Revised (SSTS-R)+ | Satisfaction with specific therapeutic process and therapist relationship | | M2 |
| System Usability Scale (SUS) | Perceived usability of information technology system (WebEx) | | M2 |
| Adverse Events+ | Safety | | M0, M3, M6, weekly during intervention |
| Off-Protocol Treatment Tracking+ | Headache or depression treatment received outside of the study | | M0, M1, M2, M3, M6, M9 |
| * Primary outcome  + Secondary outcome  † Administered only if participants indicate suicidal thoughts on the QIDS |  | |  |
|  |  | |  |
|  |  | |  |
|  |  | |  |
