# Supplementary material for: Evaluating an Abbreviated Version of Mindfulness-Based Cognitive Therapy Delivered via Telephone or Videoconferencing Compared to Enhanced Usual Care—Treatment for Migraine and Mood (TEAM-M) Study: Protocol for a Three-Arm Multisite Randomized Controlled Feasibility Trial
Source: JMIR Res Protoc. 2026 Jun 5;15:e93627. doi: 10.2196/93627 (PMC13240983; doi:10.2196/93627)
Supplement: Peer Review Report [file resprot-v15-e93627-s002.pdf]

SUMMARY STATEMENT
(Privileged Communication)

PROGRAM CONTACT:
Dr. Lanay Mudd
301-594-9346
muddlm@mail.nih.gov

Release Date: **11/13/2020**
Revised Date:

Application Number: **1 U01 AT011005-01A1**
Formerly: **1U01AT011005-01**

Principal Investigators (Listed Alphabetically):

SENG-TAMACCIO, ELIZABETH K
SHALLCROSS, AMANDA J (Contact)

Applicant Organization: NEW YORK UNIVERSITY SCHOOL OF MEDICINE

Review Group: **ZAT1 PJ (10)**
National Center for Complementary and Integrative Health Special Emphasis Panel
Exploratory Clinical Trials of Mind and Body Interventions (MB)

Meeting Date: **10/27/2020** ***RFA/PA:*** **PAR18-118**
Council: **JAN 2021** ***PCC:*** **MUDDL**
Requested Start: **04/01/2021**

Project Title: **Feasibility and Acceptability of Telephone-delivered MBCT for Migraine and Depression (FATE-MD)**
SRG Action: **Impact Score:21**
Next Steps: **Visit https://grants.nih.gov/grants/next_steps.htm**
Human Subjects: 30-Human subjects involved - Certified, no SRG concerns
Animal Subjects: 10-No live vertebrate animals involved for competing appl.
Gender: 1A-Both genders, scientifically acceptable
Minority: 1A-Minorities and non-minorities, scientifically acceptable
Age: 3A-No children included, scientifically acceptable

Project Year	Direct Costs Requested	Estimated Total Cost
1	497,237	816,813
2	498,247	818,472
3	498,143	818,301
4	497,848	817,816
5	497,518	817,274
TOTAL	2,488,993	4,088,676

ADMINISTRATIVE BUDGET NOTE: The budget shown is the requested budget and has not been adjusted to reflect any recommendations made by reviewers. If an award is planned, the costs will be calculated by Institute grants management staff based on the recommendations outlined below in the COMMITTEE BUDGET RECOMMENDATIONS section.

1U01AT011005-01A1 Shallcross, Amanda

RESUME AND SUMMARY OF DISCUSSION: This U01 resubmission application titled “Feasibility and Acceptability of Telephone-delivered MBCT for Migraine and Depression (FATE-MD)” is submitted in response to PAR-18-118 “NCCIH Mind and Body Clinical Trial Cooperative Agreement (U01)” by New York University School of Medicine with Drs. Amanda Shallcross and Elizabeth Seng-Tamaccio, as the multiple principal investigators. The overall goal of the project is to adapt a standardized, evidence-based Mindfulness-Based Cognitive Therapy (MBCT) program to a telehealth (MBCT-T) or video (MBCT-V) format, demonstrate fidelity across multiple sites, and determine feasibility/acceptability compared to Enhanced Usual Care (EUC), to reduce migraine disability and comorbid depressive symptoms. By developing an abbreviated, remote delivery intervention, this highly significant project addresses an important barrier to migraine treatment in preparation for a future clinical trial. The project leverages robust preliminary data from a prior MBCT feasibility trial to inform the current study design. The investigative team is highly productive, with complementary expertise, a history of collaboration, and a track record of successfully conducting clinical trials. Overall, the environment is strong, with adequate resources and recruitment capabilities. As one of the first nonpharmacological studies targeting migraine and depression simultaneously, this remote-delivered MBCT is highly innovative. The resubmission was responsive to previous critiques, including strengthened decision rules to inform the future clinical trial. There was thoughtful consideration for several design elements in the approach, including systematically optimized feasibility benchmarks, well-defined qualitative and quantitative measures, and adequate contingency plans for conducting a multisite study. The statistical analysis plan is strong, including well-defined migraine outcome measures and detailed decision rules. However, some minor addressable concerns were raised in the approach. The 75% completion threshold for headache diaries and the exclusion of longer follow-up measures to prepare for the next trial was not entirely well-justified. Although the EUC was considered appropriate in preparation for the next clinical trial, it was noted that it was not entirely well-matched with the intervention for time and attention and this was not adequately discussed. Concerns noted in the approach were generally considered to be minor and were outweighed by the highly significant and innovative aspects of the project, the importance of the topic under study, and the stellar qualities of the study team and the environment, yielding a very sound project with overall high impact for people with migraines and comorbid depression, thereby mitigating a significant public health concern.

DESCRIPTION (provided by applicant):
Migraine is a common, painful and highly disabling disorder. Depressive symptoms are common in people with migraine and are associated with increased suffering. Despite the significant emotional, social, and economic burden of this comorbidity, no existing treatment adequately addresses both migraine disability and elevated depressive symptoms. Development of scalable and integrative treatments that address both migraine disability and comorbid depressive symptoms has been identified as a major gap in migraine treatment. Mindfulness-Based Cognitive Therapy (MBCT) is an evidence-based and standardized treatment that is an excellent candidate for reducing disability and depressive symptoms in people with migraine. Our preliminary data show that MBCT reduced migraine-related disability; however, during exit interviews, participants noted the burden of attending 8 weekly in-person sessions was a substantial barrier to accessing care. Our preliminary data suggest an abbreviated and telephone-delivered MBCT (MBCT-T) protocol is a feasible alternative to in-person treatment. However, video- based delivery (MBCT-V) may improve patient engagement. This project aims to prepare for a definitive multi-site Phase III trial of remote-delivered MBCT-T or MBCT-V or both in patients with migraine and depressive symptoms. During Study 1, we will use guidelines from the NIH’s Behavioral Change Consortium to optimize fidelity MBCT-T and MBCT-V for patients with migraine (as defined by the International Classification of Headache Disorders – 3) and depressive symptoms (as defined by empirical cut-offs on the Patient Health Questionnaire – 9). Products developed during Aim 1 will be: training protocols, facilitator treatment manuals, fidelity checklists, MBCT-T/V Adherence and Competence Scales, fidelity coding manuals. Products will be iteratively

optimized using quantitative and qualitative fidelity metrics assessed at the patient-level (comprehension) and facilitator-level (corrective feedback, intervention delivery) in three iterations following: 1) pilot MBCT-T and MBCT-V groups led by a certified MBCT facilitator at a site in New York, NY; 2) 35-hour training for facilitators across two additional sites in the Bronx, NY and Winston-Salem, NC; and 3) pilot MBCT-T and MBCT-V groups at the two additional sites. During Study 2, we will conduct a multi-site pilot RCT (n=144) of MBCT-T, MBCT-V, and Enhanced Usual Care (EUC). We will evaluate fidelity, feasibility, and acceptability of each arm across three sites in patients with migraine and depressive symptoms. Pre-specified decision rules for fidelity, feasibility, acceptability, and clinical utility will determine the arms of the future phase III trial evaluating remotely delivered MBCT for migraine disability and depressive symptoms.

PUBLIC HEALTH RELEVANCE
Migraine is a common, painful and disabling disorder; depressive symptoms are often comorbid within people with migraine and increase suffering. The goal of this project is to evaluate the fidelity, feasibility, and acceptability of remote-delivered (telephone and video) mindfulness-based cognitive therapy (MBCT) for migraine disability and depressive symptoms. We will use pre-specified decision rules to determine study design for a future phase III clinical trial of remote-delivered MBCT.

CRITIQUE 1

Significance: 2
Investigator(s): 2
Innovation: 3
Approach: 2
Environment: 2

Overall Impact: This is a resubmission. Migraine is the world's second leading cause of disability and up to 60% up patients report accompanying depressive symptoms. This project aims to set the stage for a future Phase 3 multisite trial in migraine patients by evaluating a mindfulness-based cognitive therapy (MBCT) program delivered remotely either by telephone or video conference compared to enhanced usual medical care. The first part of this project is a multisite fidelity optimization study involving 48 patients with quantitative and qualitative feedback from both patients and facilitators. The second part of the project is a multisite pilot RCT of 144 patients randomized to either telephone or video delivered MBCT or enhanced usual care. Fidelity, feasibility, and acceptability of each arm will be evaluated across all three sites and pre-specified rules will be used to determine if both remote delivery MBCT arms will be included in the future planned RCT. The approach has many important strengths and very few weaknesses. This is a strong resubmission that has generally been responsive to the concerns raised in the previous application. It has great potential to have an important influence on the research field and clinical practice.

1. Significance:
Strengths
- Migraine is a prevalent and significant public health problem associated with major disabilities both in the US and worldwide.
- Migraine is often complicated by concomitant depression.
- There is a lack of sufficient evidence for non-pharmacological treatments for migraine.
- There is promising preliminary evidence from the investigative teams' studies of the potential benefits of mindfulness-based cognitive training (MBCT).
- The use of video-based remote delivery of MBCT will increase access to the possibility of insurance reimbursement.
- There is a good justification provided for intervention optimization.

Weaknesses
- No major weaknesses were noted.

2. Investigator(s):
Strengths
- There is a very strong investigative team with substantial and complementary trial experience and publication record relating to migraine headache and MBCT.
- There is a clear description of the multiple principal investigator plan.
- There is a good record of previous collaboration.
- There is convincing expertise at all three clinical sites.

Weaknesses
- No major weaknesses were noted.

3. Innovation:
Strengths
- This is the first project to assess non-pharmacological interventions for concurrent migraines and depression.
- Development and optimization of remote delivery of MBCT for migraines using both telephone and videoconferencing is relative novel.

Weaknesses
- No major weaknesses were noted.

4. Approach:
Strengths
- Well-defined quantitative and qualitative measures collected from both patients and facilitators have a high likelihood of leading to adequate optimization and delivery of the remote interventions.
- There is good preliminary data to inform this project.
- Feasibility, including fidelity and adherence criteria, are quantified and well-described.
- Randomization procedures are adequate.
- Outcome measures are appropriate.
- Inclusion and exclusion criteria are well described and justified.
- Recruitment and retention plans are adequate.

Weaknesses
- The relative low bar of 75% complete primary clinical outcomes at month three and the lack of long-term (1 year) follow up in a four-year project preparing for a multisite phase 3 trial are considered weaknesses.

5. Environment:
Strengths
- The multisite scientific environment in which this project will be conducted is excellent.
- The management and data collection procedures are well described.
- There are convincing institutional support letters.

Weaknesses
- No major weaknesses were noted.

Milestones:
Acceptable
- Detailed milestones are described, and adequate contingency plans are in place.

Data and Safety Monitoring:
Acceptable

Study Timeline:
Strengths

- Timelines are well described and appear attainable.

Weaknesses

- No major weaknesses were noted.

Protections for Human Subjects:
Acceptable Risks and/or Adequate Protections
Data and Safety Monitoring Plan (Applicable for Clinical Trials Only):

- Acceptable

Inclusion Plans:

- Sex/Gender: Distribution justified scientifically
- Race/Ethnicity: Distribution justified scientifically
- For NIH-Defined Phase III trials, Plans for valid design and analysis: Not applicable
- Inclusion/Exclusion Based on Age: Distribution justified scientifically

Vertebrate Animals:
Not Applicable (No Vertebrate Animals)

Biohazards:
Not Applicable (No Biohazards)

Resubmission:

- An adequate response to previous review, including criticisms, comments and suggestions.

Applications from Foreign Organizations:
Not Applicable (No Foreign Organization)

Select Agent:
Not Applicable (No Relevant Resources)

Resource Sharing Plans:
Acceptable

Authentication of Key Biological and/or Chemical Resources:
Not Applicable (No Relevant Resources)

Budget and Period of Support:
Recommend as Requested

CRITIQUE 2

Significance: 1
Investigator(s): 1
Innovation: 1
Approach: 2
Environment: 1

Overall Impact: In this resubmission, the investigators propose an RCT with three arms: 8 sessions of video-delivered MBCT (MBCT-V), 8 sessions of telephone-delivered MBCT (MBCT-T), and an 8-session education control condition ("enhanced usual care" with asynchronous weekly online educational modules). Three sites are included: NYU, Albert Einstein/Montefiore, and Wake Forest. The intervention will integrate the remotely delivered MBCT for chronic pain protocol with an in-person MBCT for migraine protocol into a remotely delivered MBCT for patients with migraine and elevated depressive symptoms. The investigators are all highly qualified, with relevant experience, and most have collaborated in the past. The institutions all have specialized headache clinics directed by co-investigators and ample resources to conduct the study. The project is innovative in that it will be the first nonpharmacological trial to target migraine and depression simultaneously and the first to systematically optimize fidelity, feasibility, and acceptability of remotely delivered MBCT. Ample pilot data suggest a high likelihood of the project's successful completion. The decision to use a health education Enhanced Usual Care condition is appropriate but not controlling for time and attention from a therapist is a weakness of this otherwise excellent project.

1. Significance:
Strengths
- Migraine is a common and disabling condition.
- Depressive symptoms can complicate the treatment of migraines, yet few investigations explicitly target this comorbidity.

Weaknesses
- The project will not enroll participants with more than moderate levels of depression, although this may be justified because MBCT is less appropriate for acute depression than for residual (presumably milder) symptoms.

2. Investigator(s):
Strengths
- Dr. Shallcross is an ND/MPH working as an Assistant Professor of Population Health and Medicine at NYU. She has a K23 to test telephone-delivered MBCT for depression in patients with hypertension and is the principal investigator on a U01 studying mindfulness and medication in hemodialysis patients.
- Dr. Minen is chief of headache research at NYU. Principal investigator of multiple studies using mobile health technology.
- Dr. Seng is a clinical health psychologist with expertise in the nonpharmacological treatment of migraine and other headache disorders. Currently, she has a K23 from the National Institute of Neurological Disorders and Stroke to develop a mobile health intervention to improve migraine management. She is an Associate Professor of Clinical Psychology at Yeshiva.
- Dr. Wells is a neurologist who completed fellowships in headache medicine and CIH at Harvard. She is a principal investigator on 3 RCTs, two specifically for headache patients, and all three using MBSR. She directs the headache program at Wake Forest.
- Dr. Lipton is a neurologist with clinical and research interests in migraine. Directs the Montefiore Headache Center. Dr. Seng's K23 mentor.
- Dr. Wang, the statistician, brings appropriate experience to the role.
- Dr. Visvanathan is a clinical psychologist who developed the telephone version of MBCT. Collaborates with Dr. Shallcross.
- Dr. Heyden develops online and multimedia tools for people with migraine; collaborates with Dr. Seng. Dr. Heyden will produce the educational material for the control condition.
- Dr. Metts has an interest in informatics, mobile health, and clinical care. He will serve as a Software Development Consultant. He has collaborated with Dr. Seng.
- There are many cross-collaborations.

Weaknesses
- No major weaknesses were noted.

2. Innovation:
Strengths
- This represents the first nonpharmacological trial to target migraine and depression simultaneously.
- This trial will be the first to systematically optimize fidelity, feasibility, and acceptability of remotely delivered MBCT. The procedures for doing so are state-of-the-art.
- The proposed intervention will integrate Dr. Shallcross' remote-delivered MBCT for chronic pain protocol and Dr. Seng's in-person MBCT for migraine protocol into a remotely delivered MBCT for migraine protocol.

Weaknesses
- No major weaknesses were noted.

4. Approach:
Strengths
- The project will compare video and telephone administration, which could provide excellent data to support a subsequent larger trial.
- The project reports a lot of relevant pilot data.
- The project includes a strong plan for piloting that will ensure that any "bugs" are worked out before the pilot RCT (2 groups of 8 first at NYU, then at other sites).
- Detailed "go-no go" decision trees are included with appropriate benchmarks.
- Outcomes mainly focus on feasibility and are appropriate and objective.

Weaknesses
- "Enhanced Usual Care" is an education control condition (8 weekly online modules) that will not control for time and attention.
- Not using a structured diagnostic interview to characterize the sample in terms of depressive and anxiety disorder diagnoses is a missed opportunity.

5. Environment:
Strengths
- NYU; Albert Einstein/Montefiore; Wake Forest; Yeshiva all have excellent facilities.
- Specialized headache treatment clinics within neurology departments at these institutions will yield the requisite sample (and clinic directors are co-investigators on the project).

Weaknesses
- No major weaknesses were noted.

Milestones:
Acceptable
- Very thorough, detailed, well-thought-out milestones.

Data and Safety Monitoring:
Acceptable
- The study will include one, but its number or composition not specified.

Study Timeline:
Strengths
- Investigators have thought clearly about what is required. Although this is a multisite trial there are resources and investigators in each site conducting similar enough work that coordination would be facilitated.

Weaknesses
- No major weaknesses were noted.

Protections for Human Subjects:
Acceptable Risks and/or Adequate Protections

- Extensive and detailed plans for handling reports of suicidality, as needed in a study that will enroll depressed individuals.

Data and Safety Monitoring Plan (Applicable for Clinical Trials Only):

- Acceptable
  - Study will include a DSMB.

Inclusion Plans:

- Sex/Gender: Distribution justified scientifically
- Race/Ethnicity: Distribution justified scientifically
- For NIH-Defined Phase III trials, Plans for valid design and analysis: Not applicable
- Inclusion/Exclusion Based on Age: Distribution justified scientifically
- The plan is to oversample men to arrive at an 86% female/14% male sample. This distribution represents the population of interest (individuals with migraines and comorbid depression).

Vertebrate Animals:
Not Applicable (No Vertebrate Animals)

Biohazards:
Not Applicable (No Biohazards)

Resubmission

- Investigators have thoughtfully responded to the previous round of reviews.
- They have increased the length of the intervention from 4 sessions to 8 sessions.
- They have made a stronger case for including a telephone condition rather than just video.
- They have greatly strengthened the decision rules, which are now a model of detail and clarity.

Budget and Period of Support:
Recommend as Requested

CRITIQUE 3

Significance: 2
Investigator(s): 2
Innovation: 2
Approach: 3
Environment: 2

Overall Impact: The application has thoughtfully considered prior feedback, resulting in a number of improvements. Strengths noted previously remain (among these being a strong investigative team, focus on a highly prevalent and debilitating condition, creative approach for addressing not only the primary medical condition but co-occurring symptoms of depression, exploration of the feasibility of two distinct telehealth delivery modes that address present patient barriers to treatment and thus, have the potential for making treatment more accessible, replication across three sites, and great care to assessing key aspects to delivering treatment in a high-quality manner). While lingering concerns center on certain aspects of the design and proposed analyses, overall, the potential for impacting clinical practice and shoring up the research base is high.

1. Significance:
Strengths

- Addressing a highly prevalent medical condition while concurrently addressing one of the most distressing psychological accompaniments of recurring headaches (i.e., depression) is a major strength.
- As the number of patients in need of treatment far exceeds those who access and complete treatment, the development of alternative delivery approaches that have the potential to minimize barriers to treatment has great potential.
- Comparing two forms of remote delivery (telephone- vs. video-based), if feasible, will provide patients with meaningful options to consider.

Weaknesses

- Little attention is devoted to efforts to identify potential predictors or factors that might favor one option over the other.
- The project focuses on a limited subset of patients with migraine (although they justify this decision based on limited prior work).

2. Investigator(s):

Strengths

- Although years of experience vary somewhat, the team is quite strong and experienced with key aspects of the proposed interventions.
- Various members have successfully collaborated on similar types of projects in the past.

Weaknesses

- No major weaknesses were noted.

3. Innovation:

Strengths

- The potential for developing and making available two effective, alternative, remote delivery models for patients seeking much-needed care is a distinct plus.
- As no currently available treatment (either in-office or remote) targets the primary condition as well as any psychological sequelae, success at doing so would constitute yet another major innovation.

Weaknesses

- Although not a major goal of this application, the incremental yield from adding the two proposed new approaches does not appear to be given much consideration.

4. Approach:

Strengths

- Extensive attention is devoted to assessing fidelity, feasibility, and acceptability in addition to clinical utility.
- A comparison to a more common pharmacological approach, but enhanced by education, is notable.

Weaknesses

- Minimal attention is given to tracking potential medication influences, changes that may happen over time, and how major medication differences may impact treatment outcome.
- Minimal information is provided to substantiate why the specific components were chosen for the enhanced UC condition, nor was mention made of how learning gains for what is taught will be assessed.
- Although comparative efficacy is not a goal of this program, mention of the likely outcome for the proposed Enhanced Usual Care (EUC) condition relative to the novel delivery modes could have been helpful in order to gain initial insights into the clinical utility of pursuing the present remote delivery options.

5. Environment:

Strengths

- The various sites included in the project all seem well-suited to the tasks proposed.

Weaknesses

- No major weaknesses were noted.

Milestones:
Acceptable

Data and Safety Monitoring:
Acceptable

Study Timeline:
Strengths

- The timeline appears reasonable, with each component clear and justified.

Weaknesses

- No major weaknesses were noted.

Protections for Human Subjects:
Acceptable Risks and/or Adequate Protections
Data and Safety Monitoring Plan (Applicable for Clinical Trials Only):

- Acceptable

Inclusion Plans:

- Sex/Gender: Distribution justified scientifically
- Race/Ethnicity: Distribution justified scientifically
- For NIH-Defined Phase III trials, Plans for valid design and analysis: Not applicable
- Inclusion/Exclusion Based on Age: Distribution justified scientifically

Vertebrate Animals:
Not Applicable (No Vertebrate Animals)

Biohazards:
Not Applicable (No Biohazards)

Resubmission

- The application has thoughtfully considered prior feedback, resulting in several improvements.

Budget and Period of Support:
Recommend as Requested

CRITIQUE 4

Overall Impact: This project aims to test fidelity and feasibility of mindfulness-based cognitive therapy delivered via telephone or videoconference (MBCT-T and MBCT-V). There are some questions about the design of the study. For Aim 1, the project mentions that if the optimal fidelity is not reached, it will conduct another pilot and/or conduct additional facilitator training. However, the optimal fidelity measure is defined for each patient, and every patient meeting the optimal fidelity criterion is not expected. In fact, the project proposes to iteratively modify the procedure to enhance fidelity based on previous feedback. So, it is not clear what it means by “if one or more of the optimal fidelity thresholds are not met”. Is it measured by the proportion of patients who reach optimal fidelity in a subgroup? For Aim 2, the purpose of investigating the feasibility of enhanced usual care (EUC) is not entirely well-justified. Patients are randomized to three arms (MBCT-T, MBCT-V, and EUC). However, it seems that the

sample size calculation for Aim 2A and 2B (feasibility and acceptability) are based on a pooled sample of the arms.

THE FOLLOWING SECTIONS WERE PREPARED BY THE SCIENTIFIC REVIEW OFFICER TO SUMMARIZE THE OUTCOME OF DISCUSSIONS OF THE REVIEW COMMITTEE, OR REVIEWERS' WRITTEN CRITIQUES, ON THE FOLLOWING ISSUES:

PROTECTION OF HUMAN SUBJECTS: ACCEPTABLE

INCLUSION OF WOMEN PLAN: ACCEPTABLE

INCLUSION OF MINORITIES PLAN: ACCEPTABLE

INCLUSION ACROSS THE LIFESPAN PLAN: ACCEPTABLE

COMMITTEE BUDGET RECOMMENDATIONS: The budget was recommended as requested.

Footnotes for 1 U01 AT011005-01A1; PI Name: Shallcross, Amanda J

NIH has modified its policy regarding the receipt of resubmissions (amended applications).See Guide Notice NOT-OD-18-197 at https://grants.nih.gov/grants/guide/notice-files/NOT-OD-18-197.html. The impact/priority score is calculated after discussion of an application by averaging the overall scores (1-9) given by all voting reviewers on the committee and multiplying by 10. The criterion scores are submitted prior to the meeting by the individual reviewers assigned to an application, and are not discussed specifically at the review meeting or calculated into the overall impact score. Some applications also receive a percentile ranking. For details on the review process, see http://grants.nih.gov/grants/peer_review_process.htm#scoring.

MEETING ROSTER

The roster for this review meeting is displayed as an aggregated roster that includes reviewers from multiple AT Special Emphasis Panels Meetings
for the 2021/01 council round.
This roster for AT is available at:
http://public.era.nih.gov/pubroster/Reports?DOCTYPE=SEP&DESFORMAT=PDF&AGENDA_SEQ_NUM_P=405232
